# Early Detection of Abnormal Prion Protein in Genetic Human Prion Diseases Now Possible Using Real-Time QUIC Assay

**DOI:** 10.1371/journal.pone.0054915

**Published:** 2013-01-25

**Authors:** Kazunori Sano, Katsuya Satoh, Ryuichiro Atarashi, Hiroshi Takashima, Yasushi Iwasaki, Mari Yoshida, Nobuo Sanjo, Hiroyuki Murai, Hidehiro Mizusawa, Matthias Schmitz, Inga Zerr, Yong-Sun Kim, Noriyuki Nishida

**Affiliations:** 1 Department of Molecular Microbiology and Immunology, Nagasaki University Graduate School of Biomedical Sciences, Nagasaki, Japan; 2 Department of Neurology and Geriatrics, Kagoshima University Graduate School of Medical and Dental Sciences, Kagoshima, Japan; 3 Department of Neuropathology, Institute for Medical Science of Aging, Aichi Medical University, Aichi, Japan; 4 Department of Neurology and Neurological Science, Graduate School, Tokyo Medical and Dental University, Tokyo, Japan; 5 Department of Neurology, Iizuka Hospital, Fukuoka, Japan; 6 Dementia Research Unit, National Reference Center for TSE, Georg-August University, Göttingen, Germany; 7 Ilsong Institute of Life Science, Hallym University, Gyeonggi-Do, Republic of Korea; 8 Global Centers of Excellence Program, Nagasaki University, Nagasaki, Japan; 9 Nagasaki University Research Centre for Genomic Instability and Carcinogenesis, Nagasaki University, Nagasaki, Japan; University of Melbourne, Australia

## Abstract

**Introduction:**

The definitive diagnosis of genetic prion diseases (gPrD) requires pathological confirmation. To date, diagnosis has relied upon the finding of the biomarkers 14-3-3 protein and total tau (t-tau) protein in the cerebrospinal fluid (CSF), but many researchers have reported that these markers are not sufficiently elevated in gPrD, especially in Gerstmann-Sträussler-Scheinker syndrome (GSS). We recently developed a new *in vitro* amplification technology, designated “real-time quaking-induced conversion (RT-QUIC)”, to detect the abnormal form of prion protein in CSF from sporadic Creutzfeldt-Jakob disease (sCJD) patients. In the present study, we aimed to investigate the presence of biomarkers and evaluate RT-QUIC assay in patients with gPrD, as the utility of RT-QUIC as a diagnostic tool in gPrD has yet to be determined.

**Method/Principal Findings:**

56 CSF samples were obtained from gPrD patients, including 20 cases of GSS with P102L mutation, 12 cases of fatal familial insomnia (FFI; D178N), and 24 cases of genetic CJD (gCJD), comprising 22 cases with E200K mutation and 2 with V203I mutation. We subjected all CSF samples to RT-QUIC assay, analyzed 14-3-3 protein by Western blotting, and measured t-tau protein using an ELISA kit. The detection sensitivities of RT-QUIC were as follows: GSS (78%), FFI (100%), gCJD E200K (87%), and gCJD V203I (100%). On the other hand the detection sensitivities of biomarkers were considerably lower: GSS (11%), FFI (0%), gCJD E200K (73%), and gCJD V203I (67%). Thus, RT-QUIC had a much higher detection sensitivity compared with testing for biomarkers, especially in patients with GSS and FFI.

**Conclusion/Significance:**

RT-QUIC assay is more sensitive than testing for biomarkers in gPrD patients. RT-QUIC method would thus be useful as a diagnostic tool when the patient or the patient's family does not agree to genetic testing, or to confirm the diagnosis in the presence of a positive result for genetic testing.

## Introduction

Prion diseases (PrD) are fatal neurodegenerative disorders characterized by the accumulation of abnormal prion protein (PrP^Sc^) in the CNS. The genetic form of human PrD (gPrD) is caused by mutations in the *prion protein gene* (*PRNP*), and is classified into genetic CJD (gCJD), Gerstmann-Sträussler-Scheinker syndrome (GSS), and fatal familial insomnia (FFI). Patients with GSS and FFI have symptoms such as dementia, dyskinesia and sleep disorders, but show no specific signal in diffusion-weighed MR imaging, and therefore the clinical discrimination of GSS and FFI from non-prion diseases such as spinocerebellar degeneration (SCA) [1] and chronic refractory sleep disorders, respectively, is problematic.

We recently developed a new *in vitro* amplification technology, designated “real-time quaking-induced conversion (RT-QUIC)”, for the detection of PrP^Sc^ in CSF of sCJD [2]. The aim of the present study was to determine whether RT-QUIC could also be of value in patients with genetic prion disease, as well as in sCJD.

## Materials and Methods

### Patients

We retrospectively analyzed 56 CSF samples obtained from gPrD patients in Japan, South Korea and Germany, including 22 cases of E200K gCJD, 20 cases of P102L GSS, 12 cases of FFI and 2 cases of V203I gCJD (Table 1). PrP-genotyping was done using genomic DNA extracted from peripheral blood leukocytes, as described previously [3]. Informed consent was obtained from patients' families and/or patients. The study protocol was approved by the Ethics Committee of Nagasaki University Hospital (ID: 10042823) and registered with the University Hospital Medical Information Network (ID: UMIN000003301).

### Real-time QUIC and analysis of 14-3-3 and t-t-tau protein in CSF samples

We analyzed all CSF samples by RT-QUIC method as previously described [2]. 14-3-3 proteins in CSF were analyzed by Western blotting and total-tau protein was measured using an ELISA kit (INNOTEST®) as previously described [3].

### Expression and purification of recombinant human PrP

Recombinant PrP, equivalent to residues 23–231 of the human PrP sequence, (codon 129 M) was expressed, refolded into a soluble form (rHuPrP-sen), and purified essentially as described previously [2]. The concentration of rHuPrP-sen was determined by measuring the absorbance at 280 nm. The purity of the final protein preparations was ≥99%, as estimated by SDS-PAGE, immunoblotting, and liquid chromatography-mass spectrometry. Circular dichroism analysis showed the conformation of rHuPrP-sen was α-helix-rich (data not shown). After purification, aliquots of the proteins were stored at −80°C in 10 mM phosphate buffer, pH 6.8.

### Real-time QUIC

We prepared reactions in a black 96-well optical bottom plate (Nunc, Rochester, NY, USA) to a final volume of 100 µl. To avoid contamination, we prepared non-infectious materials inside a biological safety cabinet in a prion-free laboratory and used aerosol-resistant tips. The final concentrations of reaction buffer components were 500 mM NaCl, 50 mM PIPES pH 7.0, 1 mM EDTA and 10 µM Thioflavin T. The rHuPrP-sen concentration was 50 µg/ml, and only freshly-thawed rHuPrP-sen was used. CSF (5 µl per well) was used to seed the RT-QUIC reactions. The 96-well plate was covered with sealing tape (Nunc 236366) and incubated at 37°C in a plate reader (Infinite M200 or F200 fluorescence plate reader; TECAN) with intermittent shaking, consisting of 30 s circular shaking at the highest speed and no shaking for 30 s, with a 2 min pause to measure the fluorescence. The kinetics of fibril formation was monitored by reading the fluorescence intensity every 10 min using 440 nm excitation and 485 nm emission and monochromators (Infinite M200) or filters (Infinite F200).

## Results (Figure 1, Table 1, 2 and 3)

**Figure 1:**
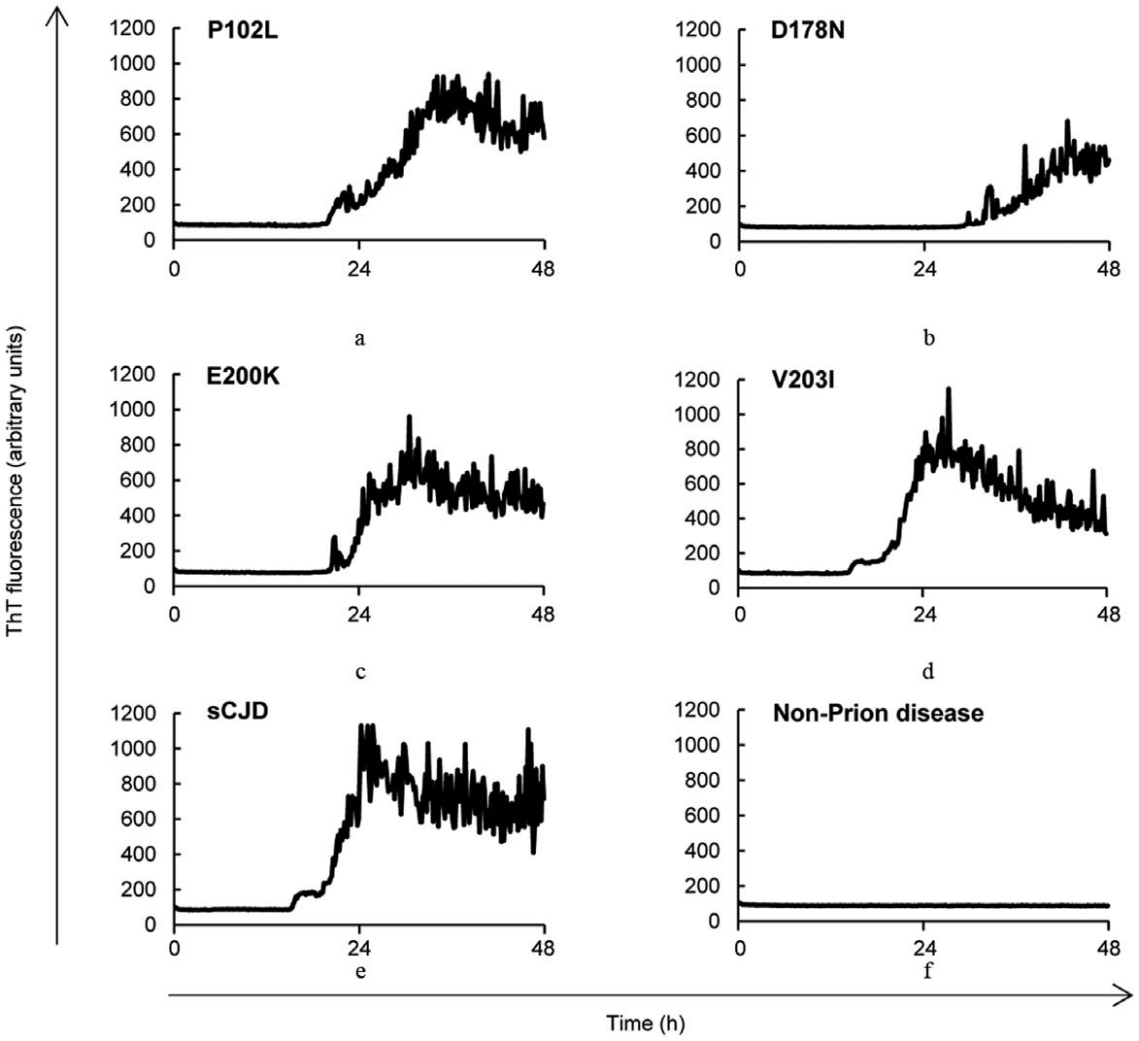
The kinetics of rHuPrP fibril formation with seeds from CSF of GSS, FFI, or gCJD. (**a**) a GSS P102L patient (**b**) a FFI D178N patient (**c**) a gCJD E200K patient (**d**) a gCJD V203I patient (**e**) a sCJD (MM1) patient and (**f**) a control subject.

First we analyzed 22 CSF samples from E200K gCJD patients. The positivities of t-tau protein, 14-3-3 protein and RT-QUIC method were all in the range of 80–85% (Figure 1, Table 1, 2 and 3). Overall, PrP^Sc^ was detected in 18 of the cases by RT-QUIC, all of which were also positive for both t-tau and 14-3-3 proteins. In the GSS and FFI cases, RT-QUIC was positive in 90% of GSS and 83.3% of FFI (Figure 1 and Table 1 and 2). Among the GSS cases, however, 80% showed negative for both t-tau and 14-3-3 proteins, and all but one of the FFI samples were negative for the biomarkers (Figure 1, Tables 1 and 2). Although we were able to analyze only 2 cases of gCJD V203I, both were positive by RT-QUIC and only one was positive for the biomarkers. All gPrD patients were methionine homozygotes at codon 129 of *PRNP*.

**Table 1:** Summary of CSF analysis of genetic prion disease cases.

	GSS	FFI	g CJD	
	P102L	D178N	E200K	V203I
Number	20	12	22	2
Age (average year)	55.5±4.45	55.8±13.7	62.7±9.43	73
Sex (m∶f)	1∶3	3∶1	1∶1	2∶0
positive patients/total (%)				
[95% CI*]				
	4/20	1/12	19/22	1/2
t-tau protein	(20%)	(8.3%)	(86.3%)	(50%)
	[2.6–37.4%]	[0–25.4%]	[70.2–100%]	
	4/20	1/12	18/22	1/2
14-3-3 protein	(20%)	(8.3%)	(81.8%)	(50%)
	[2.6–37.4%]	[0–25.4%]	[67.4–96.2%]	
	18/20	10/12	18/22	2/2
RT-QUIC	(90%)	(83.3%)	(81.8%)	(100%)
	[76.5–100%]	[70.2–100%]	[67.4–96.2%]	

* The 95% confidence interval [CI] was calculated using the adjusted Wald test, and was expressed only in groups of more than seven cases.

**Table 2:** Further analysis of CSF samples of P102L GSS patients.

	duration between the symptom onset and lumbar puncture	
	1–12 months	13–77 months
Number	8 samples	12 samples
Age (average year)	56.8±2.14	54.9±5.19
Sex (m∶f)	3∶5	2∶10
positive patients/total (%)		
t-tau protein	3/8	1/12
	37.5%	8.3%
14-3-3 protein	3/8	1/12
	37.5%	8.3%
RT-QUIC	8/8	10/12
	100%	83.3%

**Table 3:** Further analysis of CSF samples of gCJD E200K patients.

	duration between the symptom onset and lumbar puncture	
	1–3 months	4–48 months
Number	10 samples	12 samples
Age (average year)	61.4±8.96	63.8±9.67
Sex (m∶f)	7∶3	1∶2
positive patients/total patients(%)		
t-tau protein	9/10	10/12
	90.0%	83.3%
14-3-3 protein	8/10	10/12
	80.0%	83.3%
RT-QUIC	7/10	11/12
	70.0%	91.6%

We compared the kinetics of recombinant Human PrP (rHuPrP) fibril formation in CSF of sCJD patients with those of gPrD patients, and found no significant difference (Figure 1).

## Discussion

The RT-QUIC in vitro PrP^Sc^ amplification assay for diagnosis of prion disease has shown 84% sensitivity and 100% specificity in CSF samples from sporadic CJD patients.

To determine the value of RT-QUIC in genetic prion disease diagnosis, we analyzed a total of 56 CSF samples from patients with various genetic forms of human prion disease (Table 1). Our study demonstrated that RT-QUIC was highly positive in all four of the gPrD types we analyzed. Notably, most of the GSS and FFI patients were negative for both 14-3-3 and t-tau, as found in previous studies [4], whereas RT-QUIC showed 90% positivity in GSS.

RT-QUIC method was capable of detecting an extremely low volume of PrP^Sc^, and we were able to detect as little as ≥1 fg of PrP^Sc^ in diluted brain homogenate from sCJD patients. While we were not able to detect PrP^Sc^ in all sCJD CSF samples, the reasons for this are unclear and we assume that the amounts of PrP^Sc^ are very much lower in CSF samples of negative gPrD patients.

Because the majority of GSS patients remain alive with only relatively mild symptoms one year after the onset [5], many consult a clinician only later in the disease progression. On other hand, the progression of CJD is much more rapid, with most patients exhibiting akinetic mutism within 3 months. For this reason, we define the “early stage” in GSS (P102L) as 1–12 months, and in E200K gCJD as 0–3 months.

Moreover GSS and FFI show considerable phenotypic variability [6], [7], and it is very important to distinguish them from non-prion diseases at an early stage. Until now, this has not been possible. Using the RT-QUIC assay, however, we were able to confirm positivity in 100% of GSS patients at an early stage, prior to disease progression (Table 2). Thus, RT-QUIC has application in the laboratory detection of gPrD as well as sCJD, and is likely to be of particular advantage in the differential diagnosis of FFI and GSS, in which biomarkers are usually negative (Tables 2 and 3).

Interestingly, one patient with E200K gCJD was negative by RT-QUIC when sampled at 2 months after the symptom onset, but became positive when a second sample was obtain two months later. Thus, it is important that even if the CSF analysis by RT-QUIC is negative at an early stage, it should be re-examined at a later time point. Additionally, the use of RT-QUIC along with testing for the biomarkers should prove valuable for monitoring clinical trials of therapeutic agents use in gPrD patients.

We believe that RT-QUIC analysis of CSF will become invaluable in the differential diagnosis of suspected prion diseases, since not all patients with the genetic mutation go on to develop prion diseases. Alpha

In conclusion, RT-QUIC enables the early diagnosis of GSS and FFI in many patients for whom a differential diagnosis is otherwise not currently possible.
